# Characterization of human anti-V3 monoclonal antibody 904 isolated from an Indian clade C Human Immunodeficiency Virus type-1 (HIV-1) infected donor

**DOI:** 10.1186/1742-4690-10-S1-P52

**Published:** 2013-09-19

**Authors:** Raiees Andrabi, Muzamil Makhdoomi, Kalpana Luthra

**Affiliations:** 1 Department of Biochemistry, All India Institute of Medical Sciences, New Delhi, India; 2 The Scripps Research Institute, La Jolla, California, USA

## Background

Analysis of human monoclonal antibodies (mAbs) developed from HIV-1 infected donors have enormously contributed to the identification of neutralization sensitive epitopes on the HIV-1 envelope glycoprotein. We have isolated 3 anti-V3 mAbs 277, 903 and 904 by hybridoma technology from EBV transformed B cells of HIV-1 seropositive drug naive patients. The ELISA binding revealed a subtype-C and subtype-A specific binding of antibody 277 and 903 while 904 exhibited cross reactivity also with subtype-B V3. Epitope mapping of mAbs with overlapping V3 peptides showed exclusive binding to V3 crown. The antibodies displayed high and low neutralizing activity against 2/5 tier 1 and 1/6 tier 2 viruses respectively.

## Materials and methods

To identify binding specificities of mAb 904 within the V3 region specific to Indian clade C viruses, we designed synthetic V3 mutant peptides (total of 23 peptides, 13 amino acid long), by amino acid substitutions and determined the binding reactivity by ELISA. Further, to assess the binding efficiency of mAb 904 to the native viruses of different clades, intact virion binding assays were performed.

## Results

The binding of mAb 904 was substantially reduced (% binding = 15.53) by substituting with arginine-311 in place of glutamine (Q to R) (V3_3 peptide) in the crown region of the V3C. In addition to the above (Q to R), a second substitution of arginine-306 by aromatic amino acid histidine (R to H) (V3_7 peptide), almost completely abrogated the binding ability of mAb 904 with the peptide (% binding = 3.77). This revealed that glutamine and arginine at positions 311 and 306 respectively within the V3 region of envelope gp120 are crucial amino acids required for binding of 904. Lower binding potential of mAb 904 with consensus V3B as compared to V3C (V3_1 peptide) is corroborated by the low binding to V3_8 mutant peptide (same amino acid sequence as that of con V3B) (% binding = 3.55). Another residue required for binding of mAb 904 to V3 was observed to be phenylalanine (F) and when this was substituted by tryptophan (W) at position 313 of the V3 peptide (V3_16), there was substantial reduction (% binding = 15.27) in binding and when F to W change was accompanied with a second substitution of isoleucine (I) to methionine (M) at position 307 in the V3 region (V3_18), the binding of 904 to this double mutant peptide was further reduced by half (% binding =8.11). A salient finding was that mAb 904 retained the cross-reactive binding potential to V3 region of intact viruses of different clades, suggesting that V3 epitopes recognized by 904 are exposed on intact viruses.

## Conclusions

Mapping revealed that mAb 904 tolerates most subtype (C) specific changes and is a useful tool for identifying clade C epitopes for immunogen design.

